# Emerging Trend of Central Precocious Puberty: A Retrospective Study of Cases Diagnosed Before and During the COVID-19 Pandemic in a Portuguese Tertiary-Level Hospital

**DOI:** 10.7759/cureus.76504

**Published:** 2024-12-28

**Authors:** Maria Miguel Resende, Patrícia Gomes Pereira, Catarina Mendes, Maria João Oliveira, Teresa Borges, Joana Freitas

**Affiliations:** 1 Pediatric Endocrinology Unit, Department of Pediatrics, Centro Hospitalar Universitário de Santo António, Unidade Local de Saúde de Santo António, Centro Materno-Infantil do Norte Albino Aroso, Porto, PRT

**Keywords:** children, covid-19, idiopathic central precocious puberty, pandemic, precocious puberty

## Abstract

Introduction: In light of the recent evidence suggesting an increase in idiopathic central precocious puberty (ICPP) during the COVID-19 pandemic, this study aimed to assess the incidence of newly diagnosed ICPP cases and compare differences in demographic, anthropometric, and clinical characteristics pre-pandemic and during the pandemic.

Methods: We conducted a retrospective study at a national reference pediatric endocrinology unit in Portugal to evaluate the proportion of referrals for precocious puberty (PP) and, within this group, the number of ICPP cases diagnosed before (group 1: January 2018 to March 2020) and during the pandemic (group 2: April 2020 to June 2022). Additionally, we compared the demographic, anthropometric, and clinical characteristics of ICPP patients between the two groups.

Results: Out of 258 patients referred for PP, 20 were diagnosed with ICPP (19 girls and one boy). Sixteen cases were diagnosed during the pandemic, marking a significant increase (16 vs. 4, p = 0.023), especially among girls. Additionally, thelarche onset occurred earlier during the pandemic (6.9 vs. 7.3, decimal age (DA) in years, p = 0.035). Despite pandemic challenges, a prompt medical response was observed, with a shorter time from first visit to treatment (DA at the onset of treatment - DA at first visit: 0.19 vs. 0.79 years; p = 0.015). No other parameters showed significant differences. Referrals for PP during the pandemic were not significantly higher than pre-pandemic (144 of 904 vs. 114 of 758, p > 0.05).

Conclusion: This study revealed a significant four-fold increase in the diagnosis of ICPP during the pandemic, particularly among girls. Furthermore, in the pandemic group, thelarche onset was earlier, raising the question of whether increased parental presence at home contributed to heightened awareness of pubertal changes. Despite the challenges posed by the pandemic in terms of referrals and follow-up, these results point to efficient work that led to prompt treatment initiation without delays, unlike in other pathologies, as mainly observed in adults. Surprisingly, no significant increase in body mass index was observed, suggesting that other factors may be involved. To substantiate these findings and uncover additional potential contributing factors for the development of ICPP, a more extensive research effort is warranted.

## Introduction

Precocious puberty (PP) is commonly defined as the premature onset of secondary sexual characteristics before age eight in girls and nine in boys. It can be categorized into central PP, triggered by early activation of the hypothalamic-pituitary-gonadal axis, or peripheral PP, driven by hormonal stimulation independent of the hypothalamic-pituitary-gonadal axis. It is important to accurately differentiate from benign pubertal variants, including isolated premature thelarche, premature adrenarche, or premature menarche. PP is more common in girls, generally central PP of idiopathic origin. In boys, despite being less common, it is proportionally more likely to have a serious underlying cause, frequently involving central nervous system abnormalities [1-3].

A key clinical sign in girls with idiopathic central precocious puberty (ICPP) is breast development (Tanner breast stage 2 or above), and in boys, it is testicular enlargement (≥4 ml, Tanner genital stage 2 or above). Other clinical features include accelerated growth velocity, advanced bone age, and a faster rate of pubertal progression [2,3]. Additionally, females may present with ultrasound-detected enlargement of the ovaries and uterus, as well as altered uterine shape [2,4,5].

The laboratory-based initial assessment includes the evaluation of sex steroids and gonadotropins. The gold-standard biochemical diagnosis relies on evaluating basal luteinizing hormone (LH) and/or hypothalamic gonadotropin-releasing hormone (GnRH)-stimulated LH levels and LH/follicle-stimulating hormone (FSH) ratio. Hand and wrist X-rays are crucial for skeletal age evaluation, and additional considerations include neuroimaging and pelvic ultrasound [2,3].

The use of GnRH analogs (GnRHa) in treatment is beneficial for halting puberty progression, attenuating accelerated growth and bone maturation, and consequently optimizing final height [2,3,6].

The etiology of ICPP remains not fully understood. Beyond genetic factors, others such as environmental, ethnicity, international adoption, socioeconomic, nutritional status, psychological, and metabolic factors may all play roles in the development of ICPP [1,7-12]. Growing concerns have also emerged regarding exposure to endocrine disruptors during both prenatal and postnatal periods [13-15], as well as more recent considerations related to the use of electronic devices [16-18].

The COVID-19 pandemic has brought about drastic changes in lifestyle and social interactions. Measures like lockdowns, social distancing, and restrictions on daily activities have profoundly impacted societal functions, including the educational system adapting to remote learning. As a result, significant consequences emerged, such as increased screen time, exacerbated sedentary behavior, elevated and potentially unhealthy eating habits, disrupted sleep patterns, heightened psychological stress, and increased indoor activities, potentially leading to greater exposure to endocrine disruptors [18-21]. All these changes in the environment and lifestyle may have significantly influenced children's pubertal development [19-21].

Recent scientific evidence demonstrates a growing trend in increased referrals for PP cases, particularly central PP with rapid progression, during periods of lockdown [16,21-26].

In light of the recent evidence and throughout the clinical activities of a single pediatric endocrinology unit at a tertiary-level hospital in Portugal, we observed a continuous rise in the number of diagnosed cases of ICPP since the onset of the COVID-19 pandemic. This observation prompted us to investigate the proportion of children referred for PP and, within this group, the number of ICPP cases diagnosed before and during the pandemic. Additionally, we aimed to explore potential differences in demographic, anthropometric, and clinical characteristics of ICPP patients between the two groups (group 1: pre-pandemic vs. group 2: during the COVID-19 pandemic).

This study was previously presented as a meeting abstract and a poster at the European Society for Paediatric Endocrinology (ESPE) Annual Scientific Meeting on September 21, 2023.

## Materials and methods

On March 11, 2020, the WHO classified COVID-19 as a pandemic [27]. Considering the onset of the pandemic and the date of the first pediatric endocrinology appointment, the sample was divided into two groups: the pre-pandemic group (group 1), from January 2018 to March 2020 (inclusive), and the pandemic group (group 2), from April 2020 to June 2022 (inclusive). At first, an assessment was made to determine if there were significant differences in referrals for suspected PP, the total number of referrals, and the proportion of newly diagnosed ICPP cases among those PP referrals between the two groups.

Key definitions included precocious puberty, corresponding to the development of secondary sexual characteristics before eight years in females and before nine years in males. Idiopathic central precocious puberty was defined as the onset of breast development before eight years in females and testicular volume ≥4 ml before nine years in males. Testicular volume among male participants was assessed using a Prader orchidometer. In addition, testicular ultrasound was performed when needed to provide a more precise measurement of testicular volume. All participants classified as having ICPP in this study met both clinical and analytical criteria for hypothalamic-pituitary-gonadal axis activation, based on the gold standard criteria: basal LH > 0.3 IU/L and/or a GnRH stimulation test with peak LH > 3.3-5 IU/L and an LH/FSH ratio > 0.66 [2,28].

In addition to analytical data, imaging data, including hand and wrist X-rays, brain magnetic resonance imaging, and pelvic ultrasound, were analyzed from confirmed ICPP cases. Those who presented central nervous system anomalies and genetic syndromes were excluded. The Greulich and Pyle atlas was utilized for bone age assessment.

We also assessed demographic, clinical, and anthropometric characteristics from the first visits of ICPP patients in both groups. Data on treatment initiation and age at onset of treatment were the only exceptions, as these were obtained from subsequent consultations.

Continuous variables included decimal age (DA) at first visit (years), DA at thelarche onset (years), height (cm and standard deviation score (SDS)), weight (kg and SDS), body mass index (BMI) (kg/m2 and SDS), bone age (BA) (DA in years), BA minus DA (years), DA at the onset of treatment (years), mid-parental height (MPH) (cm and SDS), DA at the onset of treatment minus DA at first visit (years), and height SDS minus MPH SDS. Categorical variables included gender (feminine or masculine), nutritional status (normal/overweight/obese), Tanner stage at diagnosis (II/III/IV/V), menarche at diagnosis (yes or no), and required treatment with GnRH analogs (yes or no). Auxology® version 1.0 b 17, released in 2003 (Pfizer, New York, NY), was used to calculate all SDS.

Data were obtained from the medical records of a single pediatric endocrinology unit at a tertiary-level hospital. This study complies with the Declaration of Helsinki, and approval from the Unidade Local de Saúde de Santo António/Instituto de Ciências Biomédicas Abel Salazar Review Board was granted on September 27, 2024, (Approval number: 2024.148 (119-DEFI/131-CE)). Data were collected using pseudo-random alphanumeric identifiers, ensuring irreversible anonymization. Verbal consent for data collection and potential publication was obtained from legal guardians during each appointment. Written consent was waived by the Ethics Committee, following the appropriate rationale. Data collection, processing, and analysis were performed using the IBM SPSS Statistics 27® program (IBM Corp., Armonk, NY), ensuring that the assumptions of the applied tests were met. The number of referrals for suspected PP, within the total referrals, and newly diagnosed ICPP cases, from those suspected PP referrals, were compared between the two groups using the chi-square test. Frequency distributions were used to describe these values. The characteristics of the study population were described using frequency distributions for categorical variables, and due to the small sample size, statistical significance is not presented. The statistical significance of the continuous variable comparisons was evaluated through the Mann-Whitney test, and these were described using median and interquartile ranges (25th-75th percentile). Due to the small sample size, this test was chosen for all continuous variables. A p-value of <0.05 with a 95% confidence interval was considered statistically significant, based on two-tailed tests [29].

## Results

Out of 258 patients referred for PP, 20 were diagnosed with ICPP (19 girls and one boy). Strikingly, 16 of these diagnoses occurred after the onset of the COVID-19 pandemic (16 in 144 referrals for PP, 11.1%), marking a significant four-fold rise compared to the pre-pandemic period (four in 114 referrals for PP, 3.5%, p = 0.023), particularly among girls. As expected, the number of male cases was found to be less in our study.

Upon examination, we observed that the number of referrals for PP in the pandemic group (144 out of 904 total referrals, 15.9%) did not exhibit a statistically significant increase compared to the pre-pandemic group (114 out of 758 total referrals, 15.0%, p > 0.05).

The age at the first visit did not show a significant difference between the two groups, with group 1 presenting at 8.06 years and group 2 at 7.87 years (p > 0.05). However, the age at thelarche onset exhibited a statistically significant decrease in the pandemic group (6.9 years) compared to the pre-pandemic group (7.3 years, p = 0.035).

Furthermore, the age at treatment onset showed a trend toward earlier initiation in the pandemic group (8.04 years) compared to the pre-pandemic group (8.65 years), although this difference did not reach statistical significance (p > 0.05). However, the analysis of the time from the first visit to treatment onset revealed a statistically significant difference, indicating a shorter interval in the pandemic group (0.19 years) compared to the pre-pandemic group (0.79 years, p = 0.015).

Anthropometric measurements, including height, weight, and BMI, displayed no significant differences between the two groups. Other anthropometric and clinical parameters described in Table 1 revealed no significant differences between the study groups.

**Table 1:** Comparison of the proportion of children referred for PP and diagnosed with ICPP, as well as demographic, anthropometric, and clinical parameters at the first visit, among those diagnosed with ICPP in group 1 vs. group 2. Data are given as median (IQR – percentile) or frequency distributions. * Indicates statistically significant difference in the parameter between the two groups, p < 0.05. BA: bone age; BMI: body mass index; DA: decimal age; ICPP: idiopathic central precocious puberty; MPH: mid-parental height; NA: numbers too small to estimate significance; PP: precocious puberty; SDS: standard deviation score.

Variables	Group 1	Group 2	p-value
Total referrals, n	758	904	-
Referrals for PP, n	114	144	-
Proportion of referrals for PP, %	15.0	15.9	0.618
Precocious children with ICPP, n	4	16	-
Proportion of precocious children having ICPP, %	3.5	11.1	0.023*
Gender, n			
Female	4	15	NA
Male	0	1	
DA at first visit, years	8.06 (7.58-8.38)	7.87 (7.06-8.39)	0.508
DA at thelarche onset, years	7.3 (7.0-7.7)	6.9 (6.1-7)	0.035*
Height, cm	132.6 (126.1-139.5)	130 (125.2-137.8)	0.777
Height SDS	1.42 (0.61-2.14)	1.32 (0.55-2.04)	0.850
Weight, kg	28.75 (26-35)	29.3 (25.7-39.1)	0.962
Weight SDS	0.72 (0.14-1.55)	1 (0.25-1.73)	0.705
BMI, kg/m^2^	16.36 (16.01-18.28)	18 (15.89-20.17)	0.571
BMI SDS	0.26 (-0.07-1.07)	0.94 (0.14-1.67)	0.478
BMI status, n			
Normal	3	9	NA
Overweight	1	5	
Obese	0	2	
Tanner stage at diagnosis, n			
II	1	7	NA
III	2	7	
IV	1	1	
V	0	1	
BA, DA in years	10.50 (9.25-11)	9 (7.42-10)	0.182
BA - DA, years	2.09 (1.50-2.63)	1.25 (0.67-1.79)	0.185
Menarche at diagnosis, n			
Yes	1	1	NA
No	4	15	
Required treatment, n			
Yes	3	15	NA
No	1	1	
DA at the onset of treatment, years	8.65 (8.28-8.83)	8.04 (7.25-8.53)	0.213
MPH, cm	161 (159.3-164.7)	164 (159.2-166)	0.688
MPH, SDS	-0.18 (-0.46-0.44)	0.16 (-0.67-0.66)	0.920
Height SDS - MPH SDS	1.89 (0.33-2.45)	1.02 (0.57-1.90)	0.484
DA at the onset of treatment - DA at the first visit, years	0.79 (0.58-1.15)	0.19 (0.08-0.29)	0.015*

## Discussion

As anticipated, our study unveiled a substantial increase in the diagnosis of ICPP during the pandemic, particularly among girls.

Notably, we observed a statistically significant earlier onset of thelarche in the pandemic group. This finding raises the question of whether heightened parental presence at home during the pandemic led to increased awareness of changes in pubertal development.

Despite the challenges posed by the pandemic, particularly in terms of referrals and follow-up, our results highlight the effectiveness of our approach. This efficiency ensured the prompt initiation of treatment without delays, as demonstrated by the difference in age at treatment onset compared to the first visit in the pandemic group (0.19 vs. 0.79 years, p = 0.015).

Surprisingly, our data revealed no significant increase in BMI, suggesting that other factors beyond weight gain may be involved in the activation of the hypothalamic-pituitary-gonadal axis.

In recent studies conducted across various global centers, there is a consistent pattern aligning with our observations, indicating a worldwide surge in the diagnosis of ICPP and a concurrent increase in cases of rapidly progressive puberty [22].

The initial documentation of this phenomenon originates from Italy, where a study revealed a significant increase in newly diagnosed ICPP cases among girls during and after the Italian lockdown for the COVID-19 infection (March - July 2020), compared to the previous five years. Those diagnosed during the pandemic demonstrated an onset of puberty at a significantly younger age, accompanied by higher hormonal levels (LH, estradiol, LH level in GnRH stimulation test). Notably, this study also highlighted an accelerated pubertal progression during or after lockdown in previously diagnosed cases of slow progression ICPP. This accelerated trend was referred to as potentially linked to environmental stressors, including heightened electronic device use. Although no significant differences were found in auxological parameters among children diagnosed with ICPP before and during the lockdown, a notable finding was the significant increase in BMI among children diagnosed with slowly progressive ICPP before the lockdown, with a subsequent visit during or after the lockdown. This contrasted with the control group, where both visits occurred only before the lockdown [16].

In Italy, another study reported an increase in new ICPP cases in girls after lockdown measures, with an incidence rate of ICPP from April 2020 to April 2021 being 2.5 times higher than the period from 2017 to 2020. Similarly, this study found no significant differences in BMI between the two groups. ICPP cases diagnosed during the pandemic exhibited higher hormonal levels at diagnosis, along with increased rates of sleep disturbances and later bedtime compared to controls [21].

This emerging trend has also been emphasized in reports from Turkey [23-25]. One of these was a multi-center study where the number of ICPP cases in the pandemic group (March 9, 2020 - September 9, 2020) was also significantly higher than those in the pre-pandemic group (March 9, 2019 - March 9, 2020), associated with increased rates of treatment with GnRH analogs [28]. Another study documented an increase in ICPP and rapidly progressive early puberty female cases, which in total were approximately three times higher during the first 15 months after the onset of the pandemic (April 2020 - June 2021) compared to the same period before (January 2019 - March 2020) [24]. These reports also did not find a significant difference in BMI SDS between pre-pandemic and pandemic groups [23-25].

Another example is from India, where the study reported a three-fold increase in referrals for precocity during the COVID-19 lockdown, especially in girls. The proportion of ICPP cases during the lockdown period was significantly higher compared to the pre-lockdown period. In this study, while BMI did not show significant differences between groups, a more advanced bone age and a notable difference in bone age and chronological age in the lockdown group were observed, suggesting a potentially more rapidly progressive puberty. Additionally, though the Tanner stage at the first visit was not significantly different between the two groups, an increase in the proportion of patients presenting with late puberty was observed in the lockdown group [26].

The present study has some limitations that need to be considered. Firstly, the data were collected from a single center, resulting in a small sample size, which potentially limits the generalizability of the findings. Additionally, although there are a number of hypothetical factors that may be associated with the increased frequency of ICPP, such as lifestyle changes during the pandemic, they were not investigated in this study. Another limitation is that the data were obtained from the medical records of the first visit, with information related to follow-up limited to the initiation of treatment and age at the start of treatment. Other data points, such as the velocity of puberty progression and bone age evolution during follow-up, were not assessed or analyzed.

## Conclusions

In conclusion, our study highlights a significant rise in the diagnosis of ICPP in girls during the COVID-19 pandemic, consistent with global reports of increased ICPP and rapidly progressive puberty cases. These findings call for further long-term research to understand the contributing factors and post-pandemic trends, enabling the development of effective preventive measures to protect children's well-being.
